# P-2004. Risk Factors for Death in Children with Multisystem Inflammatory Syndrome in Children — United States, 2020-2022

**DOI:** 10.1093/ofid/ofae631.2161

**Published:** 2025-01-29

**Authors:** Anna R Yousaf, Regina Simeone, Katherine N Lindsey, Ami B Shah, Michael J Wu, Rebecca J Free, Laura D Zambrano, Angela P Campbell

**Affiliations:** Centers for Disease Control and Prevention, Atlanta, Georgia; Centers for Disease Control and Prevention, Atlanta, Georgia; Centers for Disease Control and Prevention, Atlanta, Georgia; General Dynamics Information Technology, Atlanta, Georgia; Centers for Disease Control and Prevention, Atlanta, Georgia; Centers for Disease Control and Prevention, Atlanta, Georgia; Centers for Disease Control and Prevention, Atlanta, Georgia; Centers for Disease Control and Prevention, Atlanta, Georgia

## Abstract

**Background:**

Although multisystem inflammatory syndrome in children (MIS-C) incidence has decreased, cases continue to occur and can have notable morbidity and mortality. This investigation evaluates clinical and demographic risk factors for death from MIS-C.

**Table 1.**
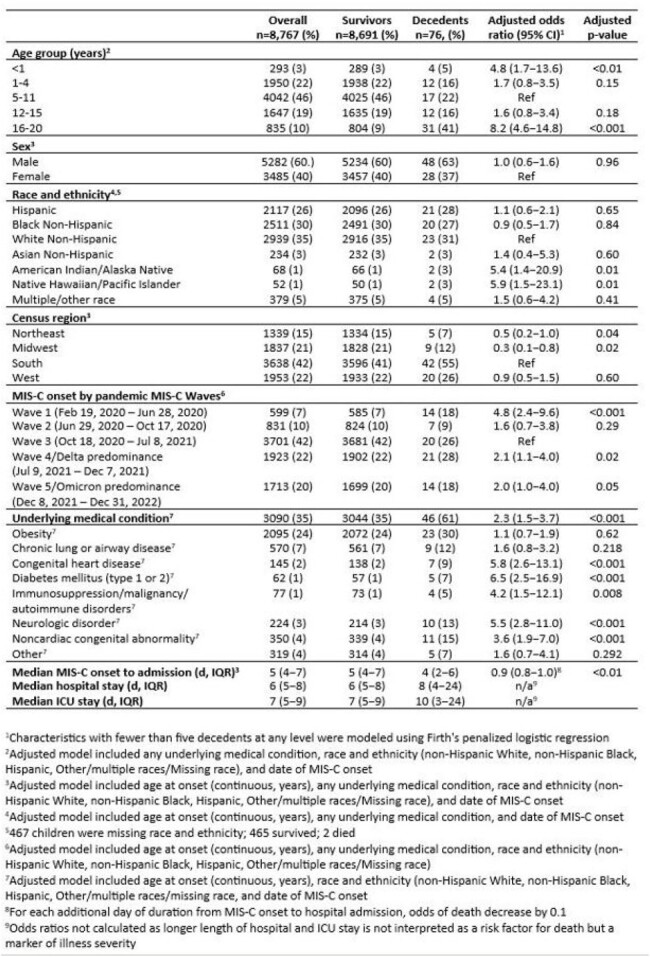
Demographic and clinical risk factors for death in children with Multisystem Inflammatory Syndrome in Children, United States, 2020-2022

**Methods:**

We evaluated children reported to CDC national MIS-C surveillance with illness onset February 2020–December 2022 and known illness outcome. We performed multivariable logistic regression to estimate odds of death compared with survival for patient characteristics and MIS-C organ involvement. Model covariates were selected *a priori* including age at MIS-C onset, date of MIS-C onset, race and ethnicity, and presence of any underlying medical condition. To compare risk of death across the pandemic we evaluated patients with date of MIS-C onset in five pandemic waves corresponding to peaks of MIS-C activity.

**Table 2.**
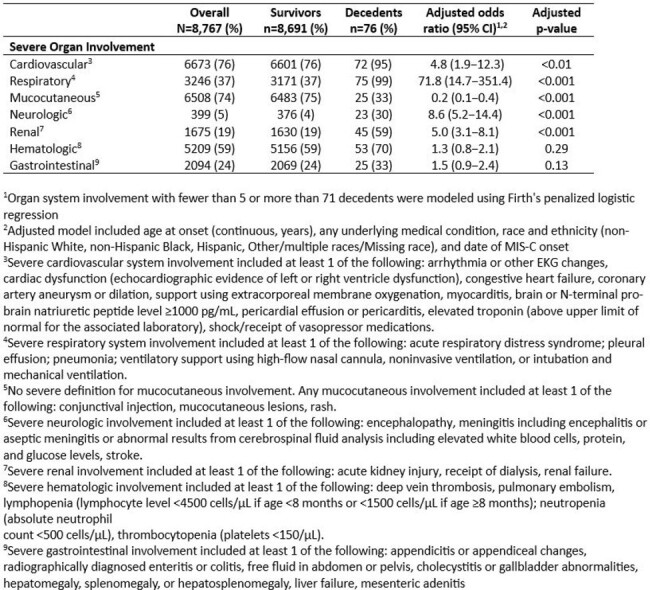
Organ system involvement risk factors for death in children with Multisystem Inflammatory Syndrome in Children, United States, 2020-2022

**Results:**

Of 8,767 MIS-C cases reported, there were 76 (9%) deaths (Table 1). Compared with children aged 5–11 years, children < 1 year and 16–20 years had increased odds of death (aOR 4.8 [1.7–13.6], p< 0.01, and 8.2 [4.6–14.8], p< 0.001, respectively). American Indian/alaska Native (AIAN) and Native Hawaiian/Pacific Islander (NHPI) children had increased odds of death compared with White non-Hispanic children (aOR 5.4 [1.4–20.9, p=0.01 and 5.9 [1.5–23.1, p=0.01, respectively). Children with MIS-C illness onset after wave 1 had decreased odds of death compared with those during wave 1. Presence of >1 underlying medical condition was associated with higher odds of death (aOR 2.3 [1.5–3.7, p< 0.001); diabetes, congenital heart disease, neurologic, immunosuppressive/autoimmune, and noncardiac congenital disorders were all independently associated with death. Cardiovascular, respiratory, neurologic, and renal MIS-C organ involvement increased odds of death while children with mucocutaneous involvement had decreased odds (Table 2).

**Conclusion:**

Younger (< 1 year) and older (16-20 years) age, AIAN and NHPI race and ethnicity, MIS-C early in the pandemic, and underlying medical conditions are risk factors for death from MIS-C. Cardiovascular, respiratory, neurologic, and renal involvement increased risk of death. Identifying risk factors associated with death may help clinicians with management and prognosis.

**Disclosures:**

Regina Simeone, PhD, Pfizer: Stocks/Bonds (Private Company)

